# Fertility induction associated with improved peripheral reproductive parameters in male *Prop1^df/df^*mice subjected to GH and levothyroxine replacement

**DOI:** 10.20945/2359-4292-2024-0086

**Published:** 2024-11-06

**Authors:** Bruna Viscardi Azevedo, Juliana Moreira Marques, Nicholas Trigueiro, Victor Yuji Yariwake, Mariana Matera Veras, Leticia Kaory Tamashiro, Robison Cruz, Luciani R. Silveira de Carvalho

**Affiliations:** 1 Hospital das Clínicas da Faculdade de Medicina da Universidade de São Paulo Laboratório de Hormônios e Genética Molecular LIM/42 Divisão de Endocrinologia São Paulo SP Brasil Hospital das Clínicas da Faculdade de Medicina da Universidade de São Paulo, Laboratório de Hormônios e Genética Molecular LIM/42 – Divisão de Endocrinologia, São Paulo, SP, Brasil; 2 Faculdade de Medicina da Universidade de São Paulo Departamento de Patologia Hospital das Clínicas São Paulo SP Brasil Hospital das Clínicas, Laboratório de Poluição Atmosférica Experimental LIM/6 – Departamento de Patologia da Faculdade de Medicina da Universidade de São Paulo, São Paulo, SP, Brasil; 3 Universidade Federal de São Paulo Divisão de Urologia Departamento de Cirurgia São Paulo SP Brasil Departamento de Cirurgia, Divisão de Urologia, Universidade Federal de São Paulo, Escola Paulista de Medicina, São Paulo, SP, Brasil; 4 Universidade de São Paulo Biotério Central da Faculdade de Medicina São Paulo SP Brasil Biotério Central da Faculdade de Medicina da Universidade de São Paulo, São Paulo, SP, Brasil

**Keywords:** Ames dwarf mice, congenital hypopituitarism, puberty, sexual maturation, pituitary

## Abstract

**Objective:**

The aim of this study was to characterize the parameters of reproductive anatomy and pituitary hormone expression levels in ames dwarf mice *(Prop1*^df/df^).

**Materials and methods:**

Male *Prop1^df/df^* mice aged 30 days received daily intraperitoneal injections of recombinant human GH and levothyroxine three times weekly for 60 days. The sexual maturation of these animals was compared with that of their wild-type (*Prop*^+/+^) and untreated (*Prop1^df/df^*) siblings.

**Results:**

The *Prop1^df/df^* treated group developed sexual maturation 2 weeks later than the *Prop*^+/+^ group and presented an increase in testicular weight, complete spermatogenesis, and enhanced LH and FSH expression. The *Prop1^df/df^* untreated group had low FSH expression and no offspring; most animals in this group did not develop sexual maturation during the study period.

**Conclusion:**

Replacement with GH and levothyroxine appeared to play a crucial role in restoring peripheral reproductive parameters and increasing pituitary hormone expression in *Prop1^df/df^* mice.

## INTRODUCTION

Ames dwarf mice (*Prop1^df/df^*) harbor a homozygous deleterious variant (p. Ser83Pro) in the *Prop1* gene, resulting in a lack of thyrotroph, lactotroph, and somatotroph differentiation and severely reduced luteinizing hormone (LH) and follicle-stimulating hormone (FSH) expression (1).

A pioneering study, published in 1965 by Andrzej Bartke, evaluated the effects of hormone replacement in Ames dwarf mice with primary deficiencies of growth hormone (GH), thyroid-stimulating hormone (TSH) and prolactin, and low LH and FSH levels (2). Replacement with GH and levothyroxine for 40 days restored fertility in these animals. Interestingly, the same hormone treatment administered to a group of female *Prop1^df/df^* mice was unable to restore the hypothalamic-pituitary-gonadal axis and this group remained infertile, similar to the untreated *Prop1^df/df^* mice (2).

According to scientific evidence, GH plays a significant role in mammals, contributing not only to growth and development but also to gonadal function. Studies by Childs and cols. (3,4) have shown a relationship between GH and gonadotropin-secreting cells in the pituitary gland. Notably, GH-binding protein antigens have been identified in pituitary cells containing LH and FSH, indicating a potential paracrine effect of GH in controlling the function of gonadotrophs (3).

In male *Prop1^df/df^* mice, bovine GH administration leads to insulin-like growth factor 1 (IGF-1) secretion and increased LH plasma levels. While LH also increases in response to gonadotropin-releasing hormone (GnRH) administration in these animals, the increase is lower than that observed in their normal *Prop1*^+/+^ counterparts. Additionally, pretreatment of *Prop1^df/df^* mice with GH before treatment with human chorionic gonadotropin (hCG) results in increased production of androstenedione and testosterone by the testes. This evidence indicates that the hypothalamic-pituitary-gonadal axis is altered in male *Prop1^df/df^* mice due to the absence of GH/IGF-1 secretion (5).

Chandrashekar and Bartke have shown that ovariectomized females from two groups of mice – *Prop1^df/df^* and bovine GH gene (bGH) transgenic mice – exhibit a significant increase in LH levels after GH and estrogen treatment followed by GnRH stimulation. This response contrasts with the one shown by animals receiving only GnRH stimulation without prior treatment, highlighting the synergistic effect of GH on GnRH and LH secretion (6).

These studies suggest that GH replacement has a role in the reproductive axis. However, a detailed molecular investigation of central and peripheral features in *Prop1^df/df^* mice remains necessary. For this purpose, the aim of this study was to characterize the parameters of reproductive anatomy and pituitary hormone expression levels in *Prop1^df/df^* mice. With this research, we seek to contribute to a deeper understanding of the role of pituitary hormone replacement in gene expression and the pathways involved in the terminal differentiation of the reproductive axis in these animals.

## MATERIALS AND METHODS

### Ethical considerations

All experimental procedures described in this study were approved by the Ethics Committee in Research of the University of São Paulo Medical School (CEUA; 1735/2021).

### Mice

Ames dwarf mice (*Prop1^df/df^*) are homozygous for the spontaneous mutation c.475C>T, p.S83P (ENSMUST00000051159). These mice were obtained from Dr. Andrzej Bartke (DF/B) and maintained at the University of Michigan under the care of Dr. Sally Camper, before being transported to the University of São Paulo.

Male *Prop1^df/df^* mice and their wild-type *Prop1*^+/+^ littermates were produced by mating heterozygous females and males in our breeding colony at the University of São Paulo. The *Prop1^df/df^* mice remained on an isogenic genetic background. All the animals were maintained under temperature- and light-controlled conditions (20-23 °C, 12-hour light-dark cycles), with access to food and water *ad libitum*.

The mice were genotyped using polymerase chain reaction (PCR) with the primers 5’ GAG CTG GGG AGA CCT AAG CTT TGC C 3’ and 5’ GCC CAG ATG TCA GGA TAC TG 3’, followed by PCR product digestion with Van91I (7).

### Pituitary hormone replacement

**Figure 1:**
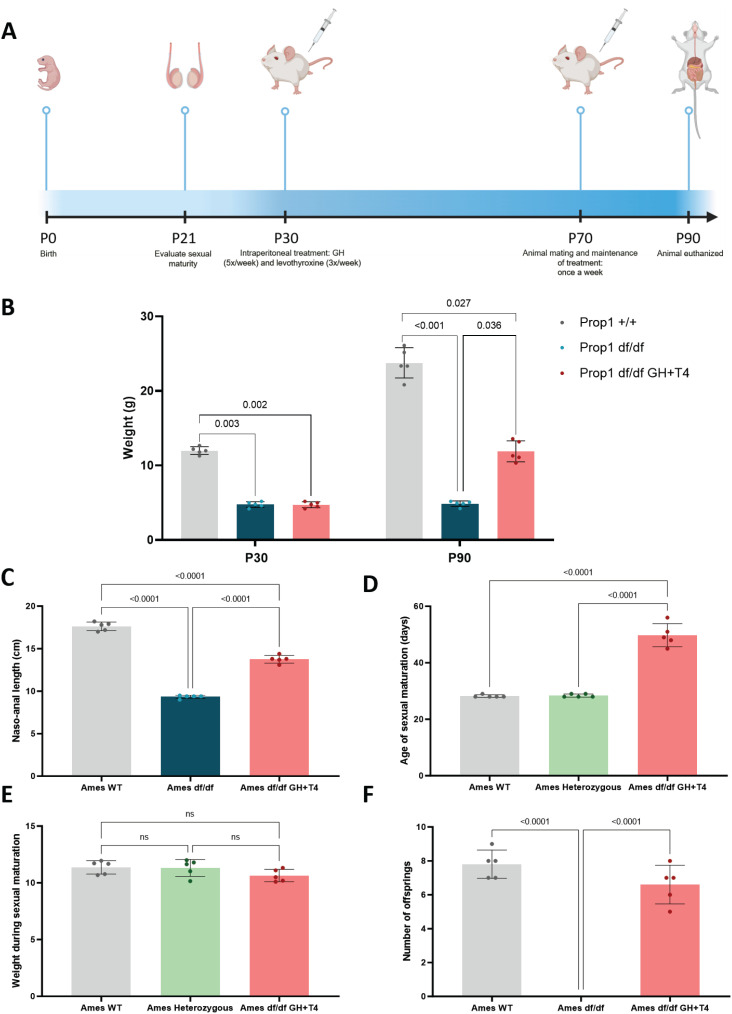
(A) Experimental timeline, shown in postnatal days (P), depicting the steps of the study. **(B)** Initial (P30) and final (P90-100) weights (in grams) in the *Prop1^+/+^*, *Prop1^df/df^*, and *Prop1^df/df^* GH+T4 groups (5 animals per group). **(C)** Comparison of naso-anal length among the groups at the experiment's conclusion (5 animals per group). **(D)** Comparison of the age of sexual maturation among the groups (in days; 5 animals per group). **(E)** Comparison of weight (in grams) at the time of sexual maturation among the groups (5 animals per group). **(F)** Comparison of the mean number of animals born per mating at the experiment's conclusion among the groups (5 animals per group). Note: The image was created with BioRender.

Male *Prop1^df/df^* mice were divided into two groups. One group (*Prop1^df/df^* GH+T4; n = 5) was treated with GH and levothyroxine, and the other group (*Prop1^df/df^*; n = 5) was treated with saline solution. The two groups were compared with their wild-type male siblings (*Prop1*^+/+^; n = 5), which were treated with saline solution and were used as controls. In brief, levothyroxine (L-thyroxine; Sigma-Aldrich, Darmstadt, Germany) was diluted in 0.9% saline solution at pH 7.8 and administered via intraperitoneal injection (2 µg per 50 µL dose) three times a week (on Mondays, Wednesdays, and Fridays) at 11:00 a.m. For GH preparation, human GH (Somatropin; Bergamo, São Paulo, Brazil) was dissolved according to the manufacturer's instructions. A 0.9% saline solution, adjusted to pH 7.8, was then added to prepare a single injection, which was administered intraperitoneally (10 µg per 50 µL dose) five times a week (Mondays, Tuesdays, Wednesdays, Thursdays, and Fridays). Hormone replacement was started 30 days after birth, continued for 6 weeks, and was followed by weekly maintenance injections until the animals were 90 days old (Figure 1A).

### Sexual maturity and fertility assessment

Verification of the sexual maturation status of the Ames (*Prop1^df/df^* GH+T4 and *Prop1^df/df^*) mice and their normal siblings (*Prop1*^+/+^ and *Prop1*^+/df^; 5 animals per each group) started at 21 days after birth and was confirmed by observing testicular descent and balanopreputial separation (Figure 1A). The mice were considered sexually mature when these two events occurred. As soon as the balanopreputial separation was observed, each mouse was individually weighed on a digital bench scale (9094 Plus, Toledo do Brasil, São Bernardo do Campo, Brazil).

After 40 days of hormone replacement (P70 in Figure 1A), each mouse was individually transferred to a litter with a *Prop1*^+/+^ female to assess if they were capable of mating and producing offspring, which was later measured as a fertility parameter (Figure 1A).

### Anthropometric measurements

Body weight was initially assessed 30 days after birth and compared with the final measurement at the end of the hormone replacement period. Additionally, both naso-anal length (in cm, measured with a ruler) and testis weight were measured at the conclusion of the treatment regimen.

### Histological analysis

After euthanasia, the animals’ abdominal cavities were carefully opened, and their testes were extracted. Excess adipose tissue was dissected, and one of the testes was fixed by immersion in a 4% paraformaldehyde solution for 24 hours. Following fixation, the tissue underwent systematic dehydration involving a progressive immersion in increasing concentrations of ethyl alcohol, before being embedded in paraffin.

The testes in paraffin-embedded blocks were sequentially sectioned into slices of precisely 5 μm thickness. The orientation of the sections followed that of the transverse axis of each organ. The sections were affixed onto glass slides and stained with hematoxylin-eosin (H&E).

The testes were categorized according to the Johnsen scoring system (Supplementary Table 1). For each animal, the classification was derived from the averaged analysis of 20 isolated seminiferous tubules. Each tubule received a numerical score ranging from 1 to 10, according to the characteristics outlined in Supplementary Table 1. The scoring for each group was determined through the average individual scores of each animal.

Random pictures of each slide were obtained for histomorphometric analysis, and 10 seminiferous tubules were measured for total area, lumen area, and cell layer height. For each animal, we sampled an average of 10 seminiferous tubules.

### Sperm parameters

The epididymis of each mouse was removed and placed in Biggers-Whitten-Whittingham (BWW) medium. Sperm was allowed to swim up into BWW for 15 min at 37 °C. A drop of sperm suspension was placed on a slide for observation of motility and for counting motile and immotile sperm cells under a light microscope (Perfect, São Paulo, Brazil). A total of 200 spermatozoa were counted for each sample on a Neubauer hemocytometer slide. Eosin dye (CI 45380, Merck KGaA, Darmstadt, Germany) was used to determine sperm viability and membrane permeability. Stained cells were considered dead cells, and unstained cells were classified as viable cells.

### Real-time polymerase chain reaction

Pituitary gland RNA was used to measure *Gh, Prl, Tshb, Lhb, Fshb*, and *Gata2* expression levels (Supplementary Table 2). First, total messenger (mRNA) was extracted from the tissue sample using TRizol reagent (Thermo Fisher Scientific, Waltham, MA, USA). The samples were quantified using a spectrophotometer (BioPhotometer; Eppendorf, Hamburg, Germany). The RNA quality and purity were assessed by 260/230 ratios in the range of 1.8-2.0, and 260/280 ratios greater than 1.6 were considered acceptable. The samples were maintained at −80 °C until further analysis.

All mRNA was reverse transcribed into cDNA using the High-Capacity cDNA Reverse Transcription Kit (Applied Biosystems, Foster City, CA, USA) according to the manufacturer's protocol.

The efficiency of the quantitative PCR (qPCR) amplification was calculated from a standard curve generated with five serial dilutions, starting at 25 ng of RNA (25 ng, 12.5 ng, 6.25 ng, and 3.1 ng), using samples from wild-type animals. Based on the data collected, a graph was plotted with the threshold cycle (CT) values versus dilution factors, and linear regression analysis was conducted to evaluate the curve slope (R^2^). Reactions exhibiting efficiencies between 90% and 110% were considered ideal, with R^2^ values close to 1 indicating a strong correlation.

The RT-qPCR was performed using SYBR Green qPCR Master Mix (Thermo Fisher Scientific) following the manufacturer's instructions. Amplifications were performed on an ABI PRISM 7000 thermal cycler (Applied Biosystems) using the following cycling conditions: 95 °C for 10 minutes, followed by 40 cycles at 95˚C for 15 seconds, and 60 °C for 1 minute. Gene expression was normalized using peptidylprolyl isomerase A (PPiA). Relative quantification was carried out using 2^-^ΔΔ^CT^ (8), and the results were converted to fold change relative to the results from age-paired wild-type mice, represented by 1. Variation was considered above the error of the method, with relative values > 2.0 or < 0.5. Each individual pituitary sample was processed in triplicate.

The results of all transcriptional analyses are presented as the average of individual values for animals in each group (3 animals per group). The values obtained from both the *Prop1^df/df^* groups and their normal siblings *Prop1*^+/+^ were normalized against those of controls.

### Statistical analysis

We first assessed the normality and homoscedasticity assumptions using the Shapiro-Wilk and Levene tests, respectively. If the data met both assumptions, we conducted an analysis of variance (ANOVA) followed by Tukey's test. If the data did not meet the assumptions, we performed a Kruskal-Wallis test followed by a Dunn's test as a *post hoc* analysis. The data were analyzed using the R environment (R Foundation for Statistical Computing, Vienna, Austria), and the graphs were generated in GraphPad Prism 8 (GraphPad Software, San Diego, CA, USA). The results were considered significant when p < 0.05.

## RESULTS

### Anthropometric measurements

#### Body weight and naso-anal length

The *Prop1^df/df^* and *Prop1^df/df^* GH+T4 groups had significantly lower weight than the *Prop1*^+/+^ group at both 30 days and 90 days (12 ± 0.46 g and 23.77 ± 2.02 g, respectively) (Figure 1B). After treatment, the *Prop1^df/df^* GH+T4 group had more weight gain (Figure 1B). Comparing the naso-anal length between animal groups at the end of the hormone replacement period, we observed greater growth in the *Prop1*^+/+^ group (17.42 ± 0.45 cm), followed by the *Prop1^df/df^* GH+T4 (13.76 ± 0.41 cm) and *Prop1^df/df^* (9.38 ± 0.2 cm) groups (Figure 1C).

### Sexual maturation and fertility

Sexual maturation occurred at the average age of 28.5 ± 0.44 days among control (*Prop1*^+/+^ and *Prop1*^+/df^) animals and 49.8 ± 4.08 days among *Prop1^df/df^* GH+T4 animals. In the untreated *Prop1^df/df^* group, none of the animals achieved sexual maturation during the 90-day period (Figure 1D). The animals’ weights on the day they became sexually mature were comparable between the *Prop1*^+/+^, *Prop1*^+/df^, and *Prop1^df/df^* GH+T4 groups (Figure 1E).

The *Prop1^df/df^* GH+T4 group exhibited fertility and demonstrated no significant difference in offspring numbers when compared with the *Prop1*^+/+^ group. Conversely, the *Prop1^df/df^* group (*i.e.*, untreated animals) remained infertile (Figure 1F).

### Testicular analysis

#### Sperm parameters

**Figure 2:**
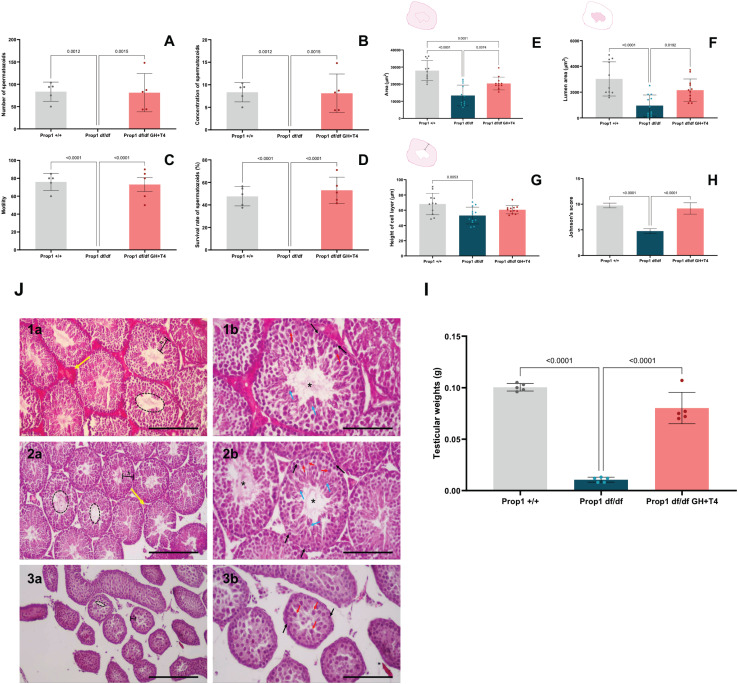
(A) Number of spermatozoa (millions/mL; 5 animals per group). **(B)** Sperm concentration (millions/mL; 5 animals per group). **(C)** Sperm motility parameter (percentage; 5 animals per group). **(D)** Sperm vitality parameter (percentage of live spermatozoa; 5 animals per group). **(E)** Analysis of total seminiferous tubule area in μm² (3 animals per group). **(F)** Analysis of lumen area in μm² (3 animals per group). **(G)** Analysis of seminiferous tubule epithelium thickness in µm (3 animals per group). **(H)** Johnsen scores in each group (3 animals per group). **(J)** Histological analyses of seminiferous tubules from animals in different groups, depicting all stages of germ cell development (3 animals per group); *Prop1^+/+^* (J1a-b), *Prop1^df/df^* GH+T4 (J2 a-b), *Prop1^df/df^* (J3 a-b).

Hormone replacement improved all spermatic parameters in the *Prop1^df/df^* GH+T4 group, including the number, concentration, motility, and survival of spermatozoa (Figure 2A-D). In contrast, the animals in the *Prop1^df/df^* group remained azoospermic (Figure 2 A-D).

#### Histological analysis

An analysis of the morphology of the seminiferous tubules showed significant improvements in the *Prop1^df/df^* GH+T4 group compared with the *Prop1^df/df^* group across all evaluated parameters (Figure 2E-G). Specifically, the lumen area and germinal epithelium size in the *Prop1^df/df^* GH+T4 group were comparable to those in the *Prop1*^+/+^ group (Figure 2F-G). Additionally, the *Prop1^df/df^* GH+T4 group had a Johnsen score (8.7 ± 0.19) higher than that in the *Prop1^df/df^* group (5 ± 0.58) but statistically comparable to that in the *Prop1*^+/+^ group (p > 0.05) (Figure 2H).

The histological analysis of the testes of the control animals (*Prop1*^+/+^) showed complete spermatogenesis, with a thick and well-organized germinal epithelium. Sertoli and Leydig cells exhibited preserved characteristics, and germ cell development was evident, including type A and type B spermatogonia cells, primary and secondary spermatocytes, rounded spermatids, and more advanced stages with elongated spermatids and abundant sperm (Figure 2J). The testes of the *Prop1^df/df^* GH+T4 animals also showed complete spermatogenesis with organized germinal epithelium and the development of germ cells according to their proximity to the tubule lumen (Figure 2J). Sertoli and Leydig cells were identified in all animals in this group (Figure 2J). In contrast, *Prop1^df/df^* animals exhibited seminiferous tubules without a lumen and with smaller diameter (Figure 2E-H), along with spermatogenesis interrupted in spermatocyte cell stages (particularly in the pachytene spermatocytes) and the presence of Sertoli cells. Leydig cells were not identified in these animals (Figure 2J).

#### Testicular weight

The testicular weight was comparable between the *Prop1^df/df^* GH+T4 (0.08 ± 0.013 g) and *Prop1*^+/+^ groups (0.1004 g ± 0.0033 g) and was lower in the *Prop1^df/df^* group (0.0166 g ± 0.0129 g) (Figure 2I).

### Molecular analysis

**Figure 3:**
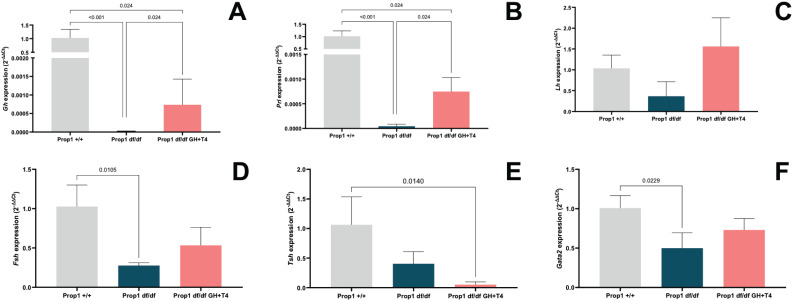
Relative expression of the genes *Gh, Prl, Lhb, Fshb, Tshb*, and the transcription factor *Gata2* in each study group (3 animals per group). The values are expressed as fold change.

After hormone replacement, the *Prop1^df/df^* GH+T4 group showed a 16-fold increase in *Gh* and *Prl* expression relative to the *Prop1^df/df^* group (1.4 × 10^−5^ ± 8.6 × 10^−6^ *versus* 4.4 × 10^−5^ ± 4.3 × 10^−5^, respectively). However, the expression levels in the *Prop1^df/df^* GH+T4 group remained 23,000-fold lower than those in the wild-type (*Prop1*^+/+^) animals (1 ± 0.3 and 1 ± 0.21, respectively) (Figure 3AB). Although no differences in *Lhb* expression were observed between the groups (Figure 3C), *Fshb* expression was lower in the *Prop1^df/df^* group (0.27 ± 0.03) compared with the *Prop1*^+/+^ group (p = 0.0105) but comparable between the *Prop1^df/df^* GH+T4 group and the other two groups (0.53 ± 0.22) (Figure 3D). Hormone replacement also reduced *Tshb* expression in the *Prop1^df/df^* GH+T4 group compared with the *Prop1*^+/+^ group (p = 0.014), while levels were comparable between the *Prop1*^+/+^ and *Prop1^df/df^* groups (Figure 3E). The expression of the transcription factor *Gata2* was significantly lower (p < 0.0229) in the *Prop1^df/df^* group compared with the *Prop1*^+/+^ group.

## DISCUSSION

This study aimed to characterize the features of reproductive anatomy and expression levels of pituitary hormones in an inbred Ames strain with and without hormone replacement, as they have not been described in the literature before. Replacement with GH and levothyroxine improved the testicular development and fertility of *Prop1^df/df^* mice. Notably, sexual maturation occurred 2 weeks later in this group compared with the *Prop1*^+/+^ group, which was probably due to the fact that hormone replacement in the *Prop1^df/df^* group started 30 days after birth. If GH and levothyroxine replacement had started earlier in *Prop1^df/df^* mice, sexual maturation would have probably developed at the same time in both groups. The isogenic Ames dwarf mice (*Prop1^df/df^*) in an inbred mating condition without hormone intervention remained infertile, with a slow development of testicular parameters. Despite the findings of the present study and those in the literature demonstrating that fertility is restored in male Ames dwarf mice after hormone treatment, the same results have not been observed in female Ames dwarf mice. Indeed, the current literature indicates that treatment using only GH and levothyroxine is not sufficient to restore fertility in these female animals. For them to achieve fertility parameters, implantation of the entire pituitary gland under the renal capsule is required (2,9).

The improvement in reproductive parameters in *Prop1^df/df^* mice was closely associated with GH replacement, since it is known that GH stimulates various aspects of gonadal function in hypophysectomized animals and in animals with hypopituitarism due to genetic mutations through its effect on the release of GnRH by the hypothalamus (10,11) and gonadotropins by the pituitary gland (12,13). Both FSH and LH act directly on Sertoli and Leydig cells, respectively, enabling them to release inhibin and testosterone, which are critical hormones for testicular development and spermatogenesis (14,15).

The main physiological effect of GH occurs through IGF-1. In mammals, GH-dependent hepatic production of IGF-1 may influence the testes to induce spermatogenesis. Moreover, IGF-1 may be produced by Sertoli and Leydig cells independent of GH, suggesting its potential role in initiating spermatogenesis. *In vitro* studies have shown that IGF-1 enhances GnRH-induced gonadotropin secretion by pituitary cells, suggesting its possible influence on neuroendocrine function and sexual maturation (10). Additionally, it is known that mice with GH receptor gene knockout (GHR-KO) exhibit delayed balanopreputial separation, a key external indicator of pubertal development in male rodents (16). Levothyroxine also plays a role in restoring reproductive function. Evidence shows that this hormone is responsible for directly stimulating the testes, aiding in the maturation and development of Sertoli cells and germ cells (17), and impacting spermatogenesis and the subsequent sexual maturation of animals (18).

The main finding of the present study was that the *Prop1^df/df^* GH+T4 group developed testicular parameters similar to those in the *Prop1*^+/+^ group. This probably occurred due to the hormone replacement increasing the secretion of gonadotropins, stimulating the testicular cells and the production of androgens and testicular IGF-1. Even though we did not measure IGF-1 levels (a potential limitation of our study), the IGF-1 effect was observed in the increased length and body weight of the treated group.

The *Prop1^df/df^* group had a low Johnsen score, with spermatogenesis interrupted in the spermatocyte phase, indicating that cell development failed to advance to the final sperm stage, a fact that was also confirmed by the lack of these cells in the spermogram. The finding of failed testicular development in this group was expected due to GH deficiency and low gonadotropin levels.

The molecular findings were consistent with the observation of failed spermatogenesis, as the *Prop1^df/df^* group had decreased *Fshb, Gh*, *Prl*, and *Gata2* expression, the latter being a transcription factor recently described as important for terminal differentiation of gonadotrophs and thyrotrophs (19). Despite no significant differences in *Lhb* and *Tshb* expression levels between the groups, these genes appeared to have decreased expression in the *Prop1^df/df^* group.

The increased *Fshb* expression in the *Prop1^df/df^* GH+T4 group was probably associated with GH replacement, as this hormone has been shown to increase gonadotropin plasma levels in transgenic mice expressing the human GH gene (5). Additionally, the GH and levothyroxine replacement may explain the low *Gh, Prl*, and *Tshb* expression levels in the *Prop1^df/df^* GH+T4 group. In mice, recombinant human GH binds to both GH and prolactin receptors, decreasing the synthesis of these hormones in the pituitary (20), while levothyroxine acts in the T4 signaling pathway, resulting in reduced production of TSH.

We observed that the pituitary glands in both the *Prop1^df/df^* and *Prop1^df/df^* GH+T4 groups had a hypoplastic and dysmorphic appearance (data not shown). This finding could be explained by the exogenous GH and levothyroxine replacement, which relieves the pituitary from producing these hormones.

The recovery of fertility observed in Ames dwarf mice is specific to the species. Although both humans and mice with the *PROP1* allelic variant have deficiencies of GH, TSH, and prolactin, all patients with this mutation seen at the endocrinology service at *Hospital das Clínicas – Faculdade de Medicina da Universidade de São Paulo* have FSH and LH deficiencies, and their reproductive axis has not been activated by hormone replacement. Indeed, the current literature indicates that hormone replacement is unable to induce fertility in humans, which is a phenomenon primarily observed in mice (1,21).

In conclusion, the primary aim of this study was to characterize the features of reproductive anatomy and expression levels of pituitary hormones in male *Prop1^df/df^* isogenic mice. We examined the role of hormone replacement with GH and levothyroxine on the animals’ pituitary-gonadal axis. The results showed that GH and levothyroxine replacement induced fertility and improved testicular parameters in these animals. Further studies are necessary to better understand this strain's central (pituitary) and peripheral (testes) contributions to fertility restoration.

## SUPPLEMENTARY TABLES

**Supplementary Table 1:** The Johnsen scoring system

Score	Histological criteria
10	Full spermatogenesis
9	Slightly impaired spermatogenesis, many late spermatids, disorganized epithelium
8	Less than five spermatozoa per tubule, few late spermatids
7	No spermatozoa, no late spermatids, many early spermatids
6	No spermatozoa, no late spermatids, few early spermatids
5	No spermatozoa or spermatids, many spermatocytes
4	No spermatozoa or spermatids, few spermatocytes
3	Spermatogonia only
2	No germinal cells, Sertoli cells only
1	No seminiferous epithelium

**Supplementary Table 2:** Primers used in the real-time polymerase chain reaction (RT-qPCR) analysis

		Pituitary hormones
Gene	Reference	Description
*Gh*	NM_008117	*Growth hormone*
*Lhb*	NM_008497	*Luteinizing hormone beta subunit*
*Fshb*	NM_008045	*Follicle-stimulating hormone beta subunit*
*Tshb*	NM_009432	*Thyroid-stimulating hormone beta subunit*
*Prl*	NM_001163530	*Prolactin*
*PPiA*	NM_0089071	*Peptidylprolyl isomerase A*
*Gapdh*	NM_0012897261	*Glyceraldehyde 3-phosphated ehydrogenase*
*Gata2*	NM_001355253	*GATA binding protein 2*
